# Transmission risk of Dirofilariosis in Portugal

**DOI:** 10.1186/1756-3305-7-S1-O16

**Published:** 2014-04-01

**Authors:** AM Alho, T Nunes, L Rinaldi, J Meireles, S Belo, P Deplazes, L Madeira de Carvalho

**Affiliations:** 1 Centro de Investigação Interdisciplinar em Sanidade Animal, Faculdade de Medicina Veterinária, Universidade de Lisboa, Portugal; 2 Unit of Parasitology and Parasitic Diseases, Department of Veterinary Medicine and Animal Productions, University of Naples Federico II, Naples, Italy; 3 Unidade de Parasitologia Médica, Instituto de Higiene e Medicina Tropical, Universidade Nova de Lisboa, Portugal; 4 Institute of Parasitology, Vetsuisse Faculty, University of Zurich, Switzerland

## 

Dirofilariosis is an important and potential fatal vector-borne canine disease, endemic in Portugal as well as in other Mediterranean countries, particularly due to its geographic and climatic conditions. Despite improved diagnostic methods and effective preventives, infections of *Dirofilaria *spp. continue to increase. Forty-one species of mosquitoes have been detected in mainland Portugal although only *Culex theileri *was found naturally infected with *Dirofilaria *larvae. Therefore, a study was addressed to assess *Dirofilaria *transmission risk in Portugal based on air temperatures, estimating the potential days with temperature values compatible with the transmission of *Dirofilaria *larvae between mosquito and reservoirs. A degree-days model based on *Dirofilaria *Development Units (DDUs) was used, considering minimum and maximum daily temperatures registered in five Portuguese meteorological stations, obtained in the platform wunderground.com. Preconditions for the model were: a threshold temperature of 14°C below which *Dirofilaria *development will not proceed in mosquitoes; 130 cumulative DDUs for larvae to reach infectivity; and a maximum life expectancy of 30 days for mosquito vectors.

DDU was evaluated in three areas of Portugal mainland - Porto (North, 41°9'0"N/8°37'0"W), Lisbon (Centre, 38°43'0"N/9°8'0"W) and Faro (South, 37°1'0"N/7°56'0"W), as well as in two different Portuguese islands in the Atlantic Ocean - São Miguel, Azores (37°44'0"N/25°40'0"W) and Madeira (32°38' 0"N/16°54'0"W), over the period from 2003 to 2013. The results show that the highest number of potential days with suitable conditions for *Dirofilaria *transmission was registered in Madeira with an average of 209.9 days/year, followed by Faro 175.2 days/year, Lisbon 163.5 days/year, Azores 140 days/year and Porto 117.2 days/year. The year 2006 was the one with a maximum number of potential transmission days (179.8) across the selected stations. During the last decade, 130 DDUs were inclusively registered from June to November in Porto and Azores Island, from April to November in Lisbon and Faro, and an uncommonly extended period from April to January in Madeira Island. An average *Dirofilaria *seasonal risk period ranged from a minimum of 5 months/year in Porto, 5.6 months/year in Azores, 6.4 months/year in Lisboa, 6.9 months/year in Faro and 8 months/year in Madeira.

These results are in accordance with the existing prevalence data and reinforce the value of geospatial tools, mapping the risk and helping to monitor and forecast future epidemiological trends, ensuring a continued surveillance and a "One Medicine-One Health" integrated approach.

## Funding

PhD research grant SFRH/BD/85427/2012; Project PTDC/SAU-SAP/113523/2009 supported by Fundação para a Ciência e a Tecnologia (FCT), Portugal.

